# Predictive factors for vaginal delivery in pregnant women with previous cesarean section: a case-control study

**DOI:** 10.61622/rbgo/2025rbgo93

**Published:** 2025-11-18

**Authors:** Gabriela Sabbatine Reis, Lucas Casagrande Passoni Lopes, Eleksandra Cibele Gusman Vendramini, Ênio Luis Damaso, Vera Therezinha Medeiros Borges

**Affiliations:** 1 Universidade Estadual Paulista Faculdade de Medicina de Botucatu Botucatu SP Brazil Faculdade de Medicina de Botucatu, Universidade Estadual Paulista, Botucatu, SP, Brazil.; 2 Universidade de São Paulo Faculdade de Medicina de Bauru Bauru SP Brazil Faculdade de Medicina de Bauru, Universidade de São Paulo, Bauru, SP, Brazil.; 3 Universidade de São Paulo Hospital de Reabilitação de Anomalias Craniofaciais Bauru SP Brazil Hospital de Reabilitação de Anomalias Craniofaciais, Universidade de São Paulo, Bauru, SP, Brazil.

**Keywords:** Pregnancy, Vaginal birth, Cesarean section, Predictive factors, Vaginal birth after cesarean

## Abstract

**Objective:**

To identify factors that predict the likelihood of a successful vaginal birth after cesarean section (VBAC).

**Methods:**

An observational, case-control study was conducted at Hospital das Clínicas, Botucatu Medical School, a tertiary referral center in Brazil. Medical records of women with singleton term pregnancies, one previous cesarean section, and a live fetus in cephalic presentation who delivered between January 2013 and December 2015 were reviewed. Participants were classified according to delivery mode: vaginal birth after cesarean section or repeat cesarean section. Maternal demographics, clinical and obstetric characteristics, and neonatal outcomes were analyzed. Associations were assessed using chi-square, and significant variables were entered into a multivariate logistic regression model.

**Results:**

A total of 653 women met the inclusion criteria. Of these, 324(49.6%) achieved a vaginal birth, and 329(50.4%) underwent a repeat cesarean section. Factors associated with a higher likelihood of vaginal birth included a history of prior vaginal delivery, cervical dilation of at least 4 cm at admission, a Bishop score of 6 or higher, spontaneous onset of labor, absence of chronic hypertension, and neonates classified as appropriate or small for gestational age. In contrast, advanced maternal age, unemployment, diabetes, and hypertensive disorders were associated with an increased likelihood of cesarean delivery.

**Conclusion:**

VBAC was more likely in women with cervical dilation of 4 cm or more at admission, a Bishop score of 6 or higher, a history of vaginal birth, spontaneous labor onset, absence of chronic hypertension, and lower neonatal birth weight.

## Introduction

In recent decades, the rate of cesarean sections has risen considerably. It is estimated that approximately one-third of women worldwide have given birth via this procedure.^(1)^ Some studies suggest that the number of cesarean sections has more than doubled in the past decade. Similarly, Brazil is experiencing a cesarean sections epidemic, with an estimated rate of (56%), equating to approximately 1.6 million surgeries per year. There is a significant discrepancy between public hospitals, where the cesarean rate is (40%), and private institutions, where it reaches nearly (90%).^(2)^ Consequently, the number of pregnant women with a history of cesarean sections has increased annually.^(1)^

As the prevalence of cesarean deliveries continues to rise, so does the occurrence of complications associated with this procedure. Compared to vaginal birth, cesarean delivery is associated with a higher risk of hemorrhage, blood transfusion, infections, and hysterectomy.^(3)^ Additionally, in subsequent pregnancies, the risks of placenta previa, placenta accreta, and uterine rupture are significantly increased.^(4)^

The likelihood of a successful vaginal birth after cesarean (VBAC) varies between (60%) and (80%).^(5)^ This probability is higher among women whose previous cesarean was performed due to non-recurring indications, such as fetal distress, meconium-stained amniotic fluid, or non-cephalic fetal presentation.^(6)^ In this population, the incidence of uterine rupture is estimated to range from (0.1%) to (0.9%), which does not justify routine elective repeat cesarean delivery.^(3,5)^

Several factors influence the probability of VBAC success, including maternal age, ethnicity, the reason for the previous cesarean, history of vaginal birth, cervical dilation, and spontaneous labor onset.^(7)^ Neonatal factors, such as lower estimated fetal weight and fetal sex, have also been identified as potential predictors of VBAC success.^(7,8)^

The American College of Obstetricians and Gynecologists states that women with a prior cesarean involving a transverse uterine incision, no history of uterine rupture, and a clinically adequate pelvis are candidates for VBAC, provided they deliver in a facility with appropriate resources, including obstetricians and anesthesiologists.^(3)^

Multiple predictive models have been developed to estimate VBAC success rates.^(9–11)^ The most widely recognized model, endorsed by the WHO, was developed by Grobman et al.^(9)^ It utilizes information available at the first prenatal visit to estimate VBAC probability based on six variables: maternal age, body mass index, ethnicity, prior vaginal delivery, previous successful VBAC, and whether the prior cesarean was indicated for a potentially recurrent condition. While this model provides valuable insights, few studies have validated its applicability across diverse populations.^(9)^

Given the increasing number of pregnant women with a history of cesarean section, investigating predictive factors for VBAC success is crucial for developing strategies to reduce cesarean rates nationwide. Therefore, this study aims to identify key factors associated with successful VBAC among women with a prior cesarean section.

## Methods

This was an observational, retrospective, analytical case-control study conducted to identify predictive factors for vaginal birth after a previous cesarean section. The research was carried out at the maternity ward of the Hospital das Clínicas, Botucatu Medical School (Universidade Estadual Paulista, UNESP), a tertiary-level teaching hospital located in Botucatu, São Paulo, Brazil. The institution serves as a regional referral center for high-risk pregnancies and provides comprehensive obstetric care, training, and research. The maternity ward is staffed by a multidisciplinary team, including obstetricians, nurses, and medical residents, and performs approximately 2,500 deliveries annually, encompassing both routine and high-risk cases.

All women who delivered at the institution between January 1, 2013, and December 31, 2015, were screened through hospital electronic records and delivery logs to identify eligible participants. Patient selection was carried out retrospectively, including all women meeting the study criteria to avoid selection bias. The study included women with a singleton pregnancy, gestational age of at least 37 weeks, a live fetus in cephalic presentation, and a history of one prior cesarean section. Exclusion criteria were a history or signs of uterine rupture, cephalopelvic disproportion, placenta previa, fetal growth restriction with abnormal Doppler studies, or elective indications for repeat cesarean section. Participants were divided into two groups according to delivery type: vaginal birth after cesarean section and repeat cesarean section. A 1:1 ratio of cases to controls was used, with no matching criteria applied.

The interval between deliveries was not consistently recorded in the medical records and, therefore, was neither used as a study variable nor as an inclusion criterion. At the Botucatu Medical School Hospital, all obstetric staff follow the same protocol for vaginal birth after cesarean: patients in spontaneous labor are eligible for trial of labor regardless of interdelivery interval, while labor induction is not performed when the interval is less than 18 months.

The primary outcome was delivery mode. Independent variables included maternal demographic factors (age, ethnicity, marital status, education level, employment status), pre-existing clinical conditions (such as hypertension and diabetes), obstetric history (number of prior vaginal deliveries before cesarean and indication for the previous cesarean), and factors related to the current pregnancy (gestational age at admission, cervical dilation, Bishop score, labor induction, and complications). Neonatal variables included birth weight classification for gestational age: small for gestational age (<p10), appropriate for gestational age (p10–p90), and large for gestational age (>p90) and neonatal sex.

Data were extracted directly from the hospital's standardized electronic medical records system and delivery logs, which are completed by the attending obstetric team at the time of care. Cervical dilation and Bishop scores were recorded by obstetricians at admission. Neonatal birth weight and gestational age were obtained from official birth registries, and growth percentiles were calculated using standardized charts. Data collection followed a structured protocol, and all variables were cross-checked for accuracy.

Maternal age was categorized as <20 years, 20–35 years, or >35 years; Bishop score was categorized as <6 or ≥6; and neonatal weight was classified into small, appropriate, or large for gestational age.

All eligible women who met inclusion criteria during the study period were included to minimize selection bias. Information bias was mitigated through standardized data collection. Potential confounders were addressed in the multivariate analysis. Sample size calculation was based on an estimated odds ratio of 1.92 from prior studies, with 95% confidence, 80% power, and a 1:1 case-to-control ratio, resulting in a minimum required sample of 590 participants (295 per group). Considering an anticipated 10% data loss, the final sample included 653 women, with 324 vaginal births and 329 repeat cesarean sections.

Categorical variables were summarized as absolute and relative frequencies, and comparisons between groups were performed using the chi-square test or Fisher's exact test, as appropriate. Variables with *p* < 0.05 in bivariate analysis, along with those deemed clinically relevant, were included in a multivariate logistic regression model to identify independent predictors of vaginal birth after cesarean section. Adjusted odds ratios (aOR) with 95% confidence intervals (CI) were calculated for each predictor. All statistical analyses were performed with SAS for Windows (version 9.4) and R (version 3.3.3). Missing data accounted for less than 5% of observations and were managed using complete-case analysis.

The study was conducted in accordance with the Declaration of Helsinki and the ethical guidelines of the Brazilian National Health Council for research involving human participants. All data were anonymized to ensure confidentiality. The research protocol was reviewed and approved by the Research Ethics Committee of the Botucatu Medical School (UNESP) under approval number 2.133.156/2017 and obtained the approval opinion under the CAAE (68191617.0.0000.5411).

## Results

A total of 653 women with a previous cesarean section met the inclusion criteria. Of these, 324(49.6%) achieved vaginal birth after cesarean (VBAC), and 329(50.4%) underwent repeat cesarean section. Table 1 presents the demographic and obstetric characteristics of the participants. Most women were between 20 and 35 years of age (508/653; 77.8%), while 44(6.7%) were younger than 20 years and 101(15.5%) were older than 35 years. The majority identified as White (535/653; 82.0%), were in a stable union (478/653; 73.2%), and reported paid employment (392/653; 60.0%). More than half had completed secondary education (344/653;52.7%), and smoking prevalence was low (61/653; 9.3%), with no difference between groups (*p* = 0.8436).

**Table 1:** Demographic and obstetric characteristics of the studied groups

Variables		Groups		p-value
		Vaginal (n=324)	Cesarean section (n=329)	
Maternal Age (Years)				
	<20 years	26(8.0)	18(5.5)	
	20 to 35 years old	264(81.5)	244(74.2)	0.0015
	>35 years	34(10.5)	67(20.3)	
Ethnicity				
	White	271(83.6)	264(80.2)	0.1283
Marital Status				
	Stable Union	242(74.7)	236(54.1)	0.3934
Profession				
	Paid work	214(66.0)	178(54.1)	0.0024
Education				
	Illiterate	4(1.2)	12(3.6)	
	Fundamental	129(39.8)	115(34.9)	0.0925
	Average	171(52.8)	173(52.6)	
	Superior	20(6.2)	29(8.8)	
Smoking		31(9.6)	30(9.1)	0.8436
Gestational age at delivery				
	Term	157(48.5)	159(48.3)	0.9738
	Post-date	167(51.5)	170(51.7)	
Previous vaginal deliveries				
	None	185(57.1)	256(77.8)	
	1	70(21.6)	46(14.0)	< 0.0001
	2 or more	69(21.3)	27(8.2)	
Cervical dilatation on admission				
	< 4 cm	117(36.1)	245(75.7)	< 0.0001
	≥ 4 cm	207(63.9)	84(25.5)	
Bishop Index				
	< 6	153(47.2)	249(75.7)	< 0.0001
	≥ 6	171(52.8)	80(24.3)	
Labor induction number		43(13.3)	120(36.5)	< 0.0001
Birth weight according to GA				
	SGA	35(10.8)	25(7.6)	
	AGA	276(85.2)	269(81.8)	0.0027
	LGA	13(4.0)	35(10.6)	
Female		155(47.8)	170(51.7)	0.3274

SGA: small for gestational age; AGA: adequate for gestational age; LGA: large for gestational age. Date expressed in numbers (percentage)

When comparing the two delivery groups, several variables showed statistically significant differences. Women older than 35 years were more common in the cesarean group (67/329; 20.3%) than in the VBAC group (34/324; 10.5%, *p* = 0.0015). Paid employment was reported more frequently among VBAC patients (214/324; 66.0%) compared with cesarean patients (178/329; 54.1%, *p* = 0.0024). A history of vaginal birth strongly influenced delivery mode: 256/329(77.8%) of cesarean patients had no previous vaginal birth compared with 185/324(57.1%) of VBAC patients, whereas two or more previous vaginal deliveries were reported by 69/324(21.3%) in the VBAC group and only 27/329(8.2%) in the cesarean group (*p* < 0.0001).

Labor admission findings also differed significantly between groups. Cervical dilation ≥4 cm was observed in 207/324(63.9%) of VBAC patients compared with 84/329(25.5%) of cesarean patients (*p* < 0.0001). A Bishop score ≥6 was recorded for 171/324(52.8%) of VBAC patients versus 80/329(24.3%) in the cesarean group (*p* < 0.0001). Labor induction was more frequently performed in the cesarean group (120/329; 36.5%) than in the VBAC group (43/324; 13.3%, *p* < 0.0001).

Regarding birth weight classification by gestational age, large-for-gestational-age (LGA) infants were significantly more common among cesarean deliveries (35/329; 10.6%) compared to VBAC deliveries (13/324; 4.0%, *p* = 0.0027). In contrast, small-for-gestational-age (SGA) and appropriate-for-gestational-age (AGA) newborns were more frequent in the VBAC group. No statistically significant differences were observed between groups for ethnicity (*p* = 0.1283), marital status (*p* = 0.3934), education level (*p* = 0.0925), gestational age at delivery (*p* = 0.9738), or neonatal sex (*p* = 0.3274).

Table 2 presents the clinical complications identified before and during pregnancy among the studied groups. Most women had no chronic or gestational comorbidities, with 278 of 324(85.8%) in the VBAC group and 232 of 329(70.5%) in the cesarean group. Chronic arterial hypertension was significantly more frequent among women who underwent cesarean delivery (46/329; 14.0%) compared with those who achieved VBAC (8/324; 2.5%, p < 0.0001). Pre-existing diabetes mellitus was also more common in the cesarean group (11/329; 3.3%) than in the VBAC group (2/324; 0.6%, p = 0.0126). Gestational complications followed a similar pattern. Hypertensive disorders, including gestational hypertension and preeclampsia, were observed in 43 of 329 women (13.0%) in the cesarean group compared with 21 of 324 women (6.5%) in the VBAC group (p = 0.0074). Gestational diabetes was present in 34 of 329 women (10.3%) undergoing cesarean delivery and 12 of 324 women (3.7%) in the VBAC group (p = 0.0002). Other clinical conditions showed no statistically significant differences between groups.

**Table 2:** Clinical complications prior to or resulting from pregnancy in the groups studied

Variables	Groups		p-value
	Vaginal (n=324)	Cesarean section (n=329)	
None	278(85.8)	232(70.5)	
CAH	8(2.5)	46(14.0)	< 0.0001
GH or PE	21(6.5)	43(13.0)	0.0074
Diabetes	2(0.6)	11(3.3)	0.0126
Gestational diabetes	12(3.7)	34(10.3)	0.0002
Heart disease	2(0.6)	4(1,2)	0.4229
Pneumopathy	6(1.8)	6(1.8)	0.9786
Thyroid disease	7(2.2)	15(4.6)	0.4646
Depression	4(1.2)	2(0.6)	0.4014

CAH: chronic arterial hypertension; GH: gestational hypertension; PE: preeclampsia. Data expressed in numbers (percentage)

Table 3 shows the indications for the previous cesarean section. Stopping dilation or descent was more frequent in the cesarean group than in the vaginal group (111/329, 33.7% vs 77/324, 23.8%; p = 0.002). Non-cephalic fetal presentation was more common in the vaginal group (48/324, 14.8%) than in the cesarean group (20/329, 6.1%; p = 0.0001). Elective was also higher in the vaginal group (46/324, 14.2%) than in the cesarean group (31/329, 9.4%; p = 0.03). Induction failure was more frequent in the cesarean group (45/329, 13.7%) than in the vaginal group (19/324, 5.9%; p = 0.0003). Hypertensive syndromes were more frequent in the cesarean group (55/329, 16.7%) than in the vaginal group (18/324, 5.5%; p < 0.0001). Others were more frequent in the vaginal group (31/324, 9.6%) than in the cesarean group (17/329, 5.2%; p = 0.031). Unknown was more frequent in the vaginal group (58/324, 17.9%) than in the cesarean group (30/329, 9.1%; p = 0.0010). Acute fetal distress did not differ between groups (27/324, 8.3% vs 20/329, 6.1%; p = 0.13).

**Table 3:** Indications for previous cesarean section in the studied groups

Indications for cesarean section	Groups		p-value
	Vaginal (n=324)	Cesarean section (n=329)	
Stopping dilation or descent	77(23.8)	111(33.7)	0.002
Non-cephalic fetal presentation	48(14.8)	20(6.1)	0.0001
Elective	46(14.2)	31(9.4)	0.03
Acute fetal distress	27(8.3)	20(6.1)	0.13
Induction failure	19(5.9)	45(13.7)	0.0003
Hypertensive syndromes	18(5.5)	55(16.7)	< 0.0001
Others	31(9.6)	17(5.2)	0.031
Unknown	58(17.9)	30(9.1)	0.0010

Data expressed in numbers (percentage)

Table 4 summarizes the indications for the current cesarean section in the cesarean group (n = 329). Acute fetal distress and meconium occurred in 107/329(32.5%). Induction failure accounted for 69/329(21.0%), and secondary arrest of dilation for 59/329(18.0%). Macrosomia was reported in 32/329(9.7%), presentation or descent stop in 22/329(6.7%), and hypertensive syndromes in 22/329(6.7%). Less frequent indications were premature placental abruption in 7/329(2.1%), anhydramnios in 3/329(0.9%), cord prolapse in 2/329(0.6%), and imminent uterine rupture in 1/329(0.3%). Maternal request was recorded in 5/329(1.5%). The three most frequent indications represented 71.5% of cesarean deliveries.

**Table 4:** Indications for current cesarean section in the Cesarean Group

Indications for cesarean section	Number of pregnant women n(%)
Acute fetal distress and meconium	107(32.5)
Induction failure	69(21.0)
Secondary stop of dilation	59(18.0)
Macrosomia	32(9.7)
Presentation descent stop	22(6.7)
Hypertensive syndromes	22(6.7)
Premature placental abruption	7(2.1)
Anhydramnios	3(0.9)
Cord prolapse	2(0.6)
Imminent uterine rupture	1(0.3)
Maternal request	5(1.5)

Data expressed in numbers (percentage)

Table 5 presents the multivariate analysis for successful vaginal delivery in the total sample of 653 women, of whom 324(49.6%) achieved VBAC and 329(50.4%) had repeat cesarean. Cervical dilatation on admission ≥ 4 cm was associated with higher odds of VBAC (OR 2.56, 95% CI 1.64–4.00; p < 0.001). A Bishop score ≥ 6 increased the likelihood of VBAC (OR 1.66, 95% CI 1.06–2.60; p = 0.03). Prior vaginal birth showed a graded effect: one previous vaginal delivery (OR 2.33, 95% CI 1.43–3.79; p < 0.001) and two or more (OR 5.03, 95% CI 2.77–9.14; p < 0.001). Non-induced labor was associated with VBAC (OR 1.80, 95% CI 1.14–2.86; p = 0.01). Absence of chronic arterial hypertension increased VBAC odds (OR 3.76, 95% CI 1.61–8.79; p ≈ 0.002). Compared with large for gestational age newborns (reference), small for gestational age was associated with higher VBAC odds (OR 4.01, 95% CI 1.54–10.43; p = 0.004) and appropriate for gestational age likewise (OR 3.47, 95% CI 1.61–7.47; p = 0.001).

**Table 5:** Multivariate analysis of independent variables associated with successful vaginal delivery

Variables		OR (95% CI)
Cervical dilatation on admission ≥ 4cm		2.56(1.64 - 4.00)
Bishop score at admission ≥ 6		1.66(1.06 - 2.60)
Number of previous vaginal deliveries		
	One	2.33(1.43 - 3.79)
	Two or more	5.03(2.77 - 9.14)
Non-induced labor		1.80(1.14 - 2.86)
Absence of Chronic Arterial Hypertension		3.76(1.61 - 8.79)
Fetal Weight According to Gestational Age		
	SGA	4.01(1.54 - 10.43)
	AGA	3.47(1.61 - 7.47)
	LGA	1

SGA: small for gestational age; AGA: adequate for gestational age; LGA: large for gestational age

## Discussion

This study identified clinical factors independently associated with successful vaginal birth after cesarean section in a tertiary referral setting with systematic access to trial of labor after cesarean. Greater cervical dilation at admission and a Bishop score of at least six were associated with vaginal birth, as were a history of previous vaginal delivery, spontaneous onset of labor, absence of chronic arterial hypertension, and newborns not classified as large for gestational age. These findings are consistent with prior evidence that places admission cervical status at the center of delivery-mode prediction. Cervical dilation has been reported as a strong single predictor of vaginal birth,^(8,13)^ and Bishop score^(15)^ thresholds in the favorable range align with increased success.^(8,16)^ Cervical readiness at admission likely reflects established labor progress and a more favorable biomechanical context for descent, which supports early risk stratification in women with a previous cesarean.

Another significant predictor identified in our study was a history of previous vaginal deliveries. A prior vaginal birth doubled the likelihood of a subsequent vaginal delivery, whereas two or more previous vaginal births increased the probability fivefold. The graded effect of parity observed here, with one prior vaginal birth increasing odds and two or more conferring the greatest likelihood, mirrors earlier reports from different populations,^(5,17)^ although null associations have also been described.^(7)^ Because of the retrospective design and the structure of records, it was not possible to determine whether the previous vaginal birth preceded or followed the cesarean, a limitation also noted elsewhere.^(13)^

Spontaneous labor was independently associated with higher odds of vaginal birth, whereas induction was linked to lower success and frequently culminated in repeat cesarean. These findings are directionally consistent with prior reports that document reduced VBAC success when induction is required in women with a uterine scar.^(5,7,17)^ Two mechanisms likely contribute. First, confounding by indication and cervical readiness: women selected for induction more often presented with an unfavorable cervix, reflected here by lower Bishop scores and smaller cervical dilatation at admission, both of which were themselves independent predictors of failure. Second, the physiological advantage of spontaneous labor, which tends to progress with more efficient uterine activity and better fetal tolerance than pharmacologically stimulated labor in scarred uteri.^(18,19)^ At this institution, cervical ripening and induction followed a uniform protocol (Foley catheter for unfavorable cervix followed by oxytocin), which reduces variability in technique but may still entail a higher threshold for success than spontaneous labor. The interdelivery interval was inconsistently recorded and was not analyzed; however, induction is not undertaken when the interval is under 18 months per local policy, a safeguard aligned with safety concerns reported in the literature.^(5,7,17)^ Together, these data support counseling that prioritizes expectant management when maternal–fetal conditions allow, careful selection for induction with explicit success and stop criteria, and transparent discussion of the modest but measurable reduction in VBAC probability associated with induction.

Maternal comorbidity influenced outcomes. Chronic arterial hypertension was associated with higher cesarean frequency, plausibly reflecting clustering of cardiometabolic risks, earlier indicated deliveries, and a lower probability of entering spontaneous labor. These observations are compatible with models and reports linking maternal age, body mass index, and metabolic disorders to cesarean delivery.^(7,9)^ Anthropometric data were incomplete in this dataset, precluding robust adjustment for body mass index, an acknowledged limitation that should be addressed prospectively.

Neonatal size relative to gestational age showed the expected gradient. Large-for-gestational-age newborns were associated with cesarean, whereas small- and appropriate-for-gestational-age categories were associated with higher vaginal-birth odds, consistent with prior work that higher birth weight reduces vaginal-birth probability while macrosomia remains difficult to predict clinically.^(17)^ There was no difference by neonatal sex in this cohort, unlike findings reported elsewhere.^(7)^

The interpretation of findings regarding trial of labor after cesarean (TOLAC), defined as a planned attempt at vaginal delivery in a woman with a previous cesarean, must be considered in the context of Brazil's high cesarean section rates. TOLAC is widely recognized as a key strategy to reduce unnecessary repeat cesarean deliveries and their associated risks, including placenta accreta spectrum, surgical complications, and long-term maternal morbidity.^(20,21)^ Evidence consistently supports that, when strict eligibility criteria, standardized protocols, and adequate intrapartum monitoring are applied, TOLAC is both feasible and safe.^(20–22)^ Reported success rates are high in tertiary centers with appropriate infrastructure, often reaching 70–80% in carefully selected populations,^(22,23)^ The absence of uterine rupture in this study aligns with large datasets reporting a risk of symptomatic rupture of less than 1%, particularly in facilities with rigorous screening and emergency preparedness.^(20–25)^

Expanding access to TOLAC and providing individualized counseling based on validated predictors, such as previous vaginal delivery, maternal age, and cervical readiness, are critical to optimizing outcomes.^(20,22,24)^ Evidence indicates that institutional factors—including teaching hospital status, availability of obstetric anesthesia, and neonatal intensive care support—are positively associated with both the utilization and success of TOLAC.^(23)^ Quality improvement initiatives, such as structured care pathways, standardized clinical guidelines, and multidisciplinary training, have successfully increased both TOLAC attempts and vaginal birth after cesarean (VBAC) rates without compromising maternal or neonatal safety.^(25)^

These findings emphasize the urgent need for health systems, especially in high-cesarean settings like Brazil, to prioritize structured implementation of TOLAC as part of cesarean reduction strategies. Investment in prenatal counseling, team training, and resource allocation is essential to ensure that VBAC is offered safely and effectively. With appropriate protocols and institutional readiness, TOLAC can be a safe, evidence-based alternative to elective repeat cesarean delivery, contributing to improved maternal health outcomes and reduced surgical morbidity.^(20–25)^

Strengths include consecutive inclusion over three years in a single tertiary center with uniform protocols, use of routinely collected data with predefined variables, and multivariable adjustment for confounders. Limitations include the retrospective design, incomplete capture of interdelivery interval and anthropometrics, and the inability to classify the sequence of prior vaginal birth relative to cesarean. Induction methods were not contrasted beyond the institutional pathway, limiting external comparisons of technique-specific effects. These constraints are addressed transparently, in line with STROBE guidance.

## Conclusion

In this cohort, cervical dilation and Bishop score at admission, spontaneous onset of labor, history of prior vaginal birth, absence of chronic arterial hypertension, and newborns not classified as large for gestational age were strongly associated with successful VBAC. These findings highlight the importance of structured triage at hospital admission, prioritizing cervical assessment and readiness for labor, as well as the cautious selection and standardization of induction protocols with explicit criteria for failure. Counseling strategies should be individualized, incorporating maternal obstetric history and comorbidities to support informed decision-making. The results support the implementation of evidence-based policies to expand TOLAC in tertiary centers, particularly in countries with high cesarean rates, as part of strategies to reduce repeat cesareans and their associated risks. Future research should include prospective validation of predictive factors, incorporation of complete interdelivery interval and maternal body mass index data, comparative studies of induction and cervical ripening methods, and extended follow-up of maternal and neonatal outcomes to enhance patient safety and optimize clinical protocols.
